# A study on sheep farming practices in relation to future production strategies in Bensa district of Southern Ethiopia

**DOI:** 10.1007/s11250-017-1509-z

**Published:** 2018-01-09

**Authors:** Hizkel Kenfo, Yoseph Mekasha, Yosef Tadesse

**Affiliations:** 1 Southern Agricultural Research Institute (SARI), P.O.Box 6, Hawassa, Ethiopia; 2 LIVES, International Livestock Research Institute (ILRI), Hawassa, Ethiopia; 3 School of Animal and Range Sciences, Haramaya University (HU), P.O.Box 138, Dire Dawa, Ethiopia

**Keywords:** Bensa, Indigenous sheep, Production system

## Abstract

The study was carried out in Bensa district of Sidama zone, Southern Ethiopia. Agro-ecologically, the study sites were classified into highland and mid-altitude. The objective of the study was to identify sheep farming practices in relation to future production strategies in the study area. A total of 128 households from four kebeles (lower administrative structure) were selected purposively based on sheep population and production potential and accessibility. Data was collected through semi-structured questionnaire, focus group discussions, and key informants. The result showed that most of the household heads were male (92.75%) and mixed crop-livestock system was the dominant production system. Among the livestock species, sheep accounted for the largest proportion across the two agro ecologies and the average sheep flock size/household was 4.6 ± 0.33 and 22 4.3 ± 0.213 in highland and in mid-altitude, respectively. The primary reason of keeping sheep was for cash income and saving across the two agro ecologies. The major feed resources for sheep during the wet and dry seasons were natural pasture and crop residues respectively across the two agro ecologies. Feed shortages, disease, parasite prevalence, and market were the major sheep production constraints in highland while feed shortage, genotype, disease, parasite prevalence, and market in mid-altitude. It can be concluded that for enhancing future production from sheep in the area, emphasis is to be given on feed availability, disease management, breeding policy, and marketing *strategies.*

## Introduction

Sheep production is a major component of livestock farming in Ethiopia. About 31–38% and 21–33% of the Ethiopian smallholder farmers own sheep and goat, respectively (Asfaw and Jabbar 2008). In 2009, the official estimate of the livestock contribution to agricultural GDP was slightly more than 32 billion Ethiopian birr or $3.2 billion US dollars (IGAD 2013). Small ruminants account for about 40% of the cash income earned by farm households, 19% of the total value of subsistence food derived from all livestock production, and 25% of total domestic meat consumption (Adane and Girma 2008). Sheep contributes close to 30% of the total ruminant livestock meat output and 14% of the total domestic meat production (Workneh et al. 2004). The sheep enterprise in the Ethiopian highland, where crop and livestock production are integrated, is the most important form of investment and cash income and provides social security in bad crop years.

Local breeds contribute a cross-breed genetic diversity to global animal genetic resources (AnGR). Unfortunately, many local breeds have a small population size which puts them at risk of extinction, according to the FAO (2013) system of categorization. The study by Helen et al. (2015) in Ethiopia explained that significant difference among production systems in most sheep production and reproduction parameters was observed and this indicates the need for specific interventions with respect to the production systems. As the authors concluded the relatively large sheep flock size and higher contribution of sheep to the livelihood suggests that introduction of carefully planned and pertinent genetic improvement strategy through the involvement of the community is likely to have good chances of success. Another study by FAO (2013) indicated that application of a series of genetic tools, along with close cooperation with breeders and utilization of other tools such as innovative product marketing, may allow small breeds to not only survive, but also thrive.

Bensa district is one of the PLDs (Pilot Learning district) of Livestock and Irrigation Value Chain of Ethiopian Smallholders (LIVES) project of ILRI in Sidama highlands of Southern Ethiopia. It is one of the leading districts in terms of sheep population from Sidama zone. However, a few efforts were made to characterize the indigenous sheep production system of the district. Hence, the objective of the study was to identify sheep farming practices in relation to future production strategies in the study area.

## Materials and methods

### Description study of the area

This study was conducted in Bensa district of Sidama zone in Southern Nations Nationalities and Peoples’ Region (SNNPR) of Ethiopia. Bensa district is one of the 19 districts in Sidama zone that extends into the Oromia region of Bale Zone or Borana-like peninsula. Bensa district is bordered on the south and north by the Oromia Region, with Bona Zuria on the west, Arbegona district on the northwest, Chere district on the east, and Aroresa district on the southeast. Daye, the capital of Bensa district, is located at 420 km southeast of Addis Ababa and 135 km northeast of Hawassa city, the SNNPR capital city.

Bensa district is located at altitude which ranges from 1452 to 3129 m above sea level (m.a.s.l.). The two rainy seasons are the *belg* (short rainy season), which covers from late February to May, and the *kremt* (main rainy season), which extends from late June to early October. The average annual rainfall of the area is 1208.5 mm. The average annual temperature of the district is 19 ^ο^C. The district has three major agro ecologies where 50, 36, and 14% of moist weyna dega (mid-altitude), moist dega (highland), and moist kola (lowland) (LIVES 2012).

**Fig. 1:**
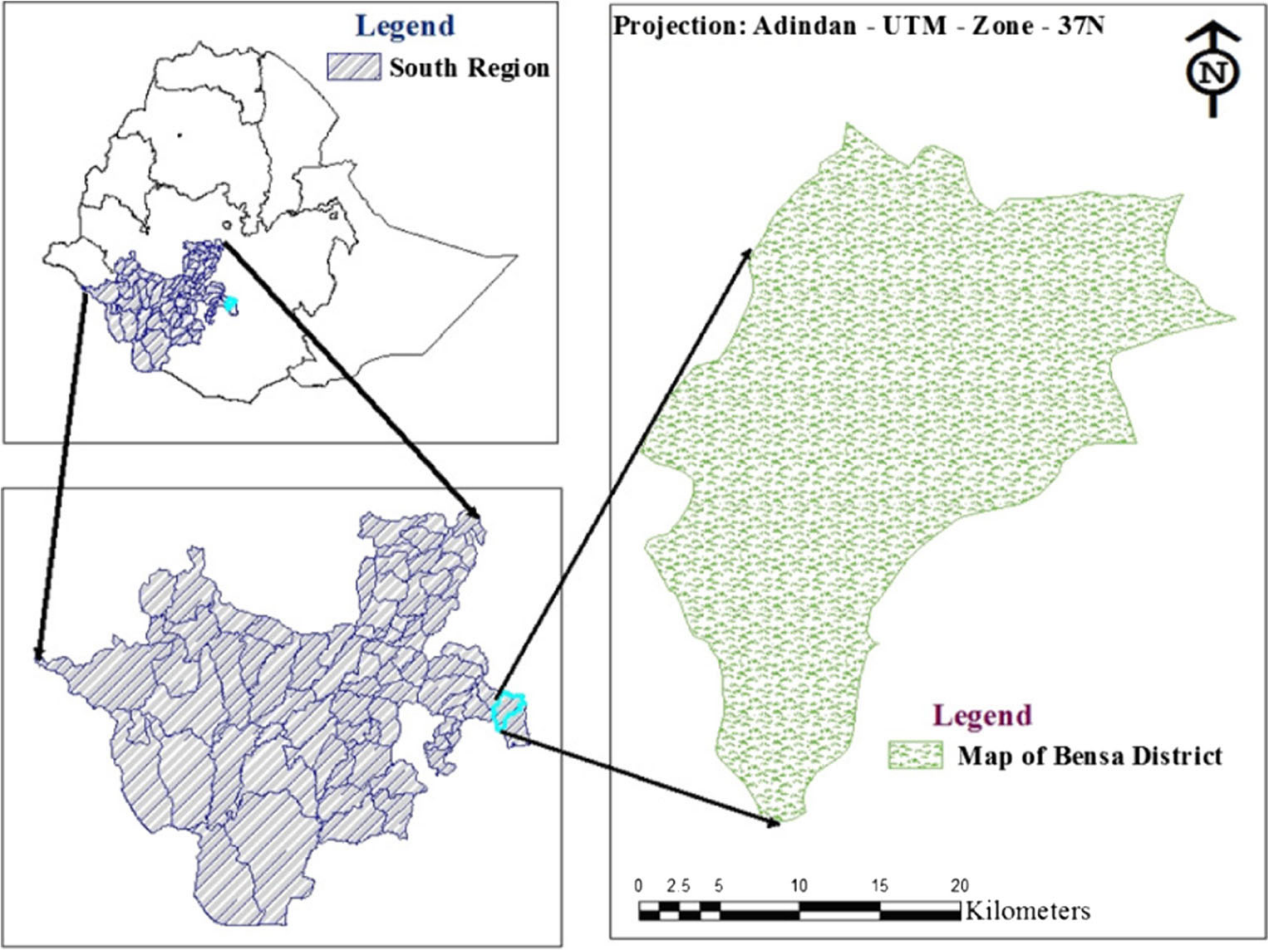
Location of the study area

The district has a population size of 342,545, of which 147,471 are men and 195,074 women. The district has an estimated numbers of 377,867, 124,021, 25,852, 231,081, 12,377, 2474, and 870 cattle, sheep, goat, chicken, horse, donkey, and mule, respectively (Bensa District of Livestock and Fishery Office 2015). Geographical location of the study area is indicated in Fig. 1.

### Sampling techniques

Bensa district was selected purposively based on the high activities of livestock and irrigation commodities including sheep by the Livestock and Irrigation Value Chain for Ethiopian Smallholders (LIVES) project (www.lives-ethiopia.org). Before deciding on the target kebeles, a preliminary survey discussions were held with the district experts, development agents, elders, and the farmers’ representatives about the local sheep types and the current production systems of the study area. The district was stratified into two categories based on sheep-dominant agro ecologies (highland and mid-altitude). From each agro ecology, two rural kebeles were selected based on the sheep population and accessibility for transportation. Then after, the list of households with minimum number of two sheep and had prior experience in sheep production was collected. This was followed by random selection of 32 households from each rural kebele. The total numbers of randomly selected sampled households were 128 from the study area.

### Data collection

Data were collected by administering a semi-structured questionnaire, individual interview, and field observations and organizing group discussions and from secondary sources. The study was undertaken from December 2015 to June 2016.

### Questionnaire and focus group discussion

A modified questionnaire was prepared by adopting a questionnaire prepared by ILRI (International Livestock Research Institute) for survey of livestock population. It was used to collect information on socio-economic characteristics, reproductive performances (age at first lambing, lambing interval, litter size, and lambing pattern), flock structure, feeds and feeding management, disease prevalence, and production constraints. Sets of open-ended questions were used to guide focus group discussions.

One key informant discussion was held with representatives of farmer groups, elders, and development agents from four kebeles. The main points for discussion included indigenous knowledge of the community about history and introduction of existing sheep type in the study area, distinctive features of the sheep type as well as their desirable and undesirable characteristics, utility and status of the existing sheep population, and other information related to indigenous sheep in the study area. Semi-structured questionnaires were also administered to the respondents of the study area.

### Data managements and analysis

The collected data was checked for any inconsistency and analyzed using SPSS (2009). Indices were calculated to provide ranking of the reasons of keeping sheep, importance of major farming activities to the family food source and income, selection criteria, and major constraints of sheep production according to the following formula: Index = Σ of [3 for rank 1 + 2 for rank 2 + 1 for rank 3] given for particular qualitative variables divided by Σ of [3 for rank 1 + 2 for rank 2 + 1 for rank 3] for all qualitative variables considered.

## Results and discussions

### Household characteristics and socio economic aspects

Household characteristics of the sampled households are presented in Table 1. The majority of the interviewed households in the study area were male headed. The age of the majority (57.05%) of the respondents who are the main source of farm labor were less than 40 years, which is the active age group.

**Table 1:** Household characteristics of the sampled households in the study area

Variables	Agro ecology					
	Highland (*n* = 64)		Mid-altitude (*n* = 64)		Overall total (*n* = 128)	
	*N*	%	*N*	%	*N*	%
Sex structure						
Male	64	100	55	85.9	119	92.95
Female	–	–	9	14.1	9	7.05
Age structure						
< 31	10	15.63	8	12.5	18	14.05
31–40	32	50	23	35.9	55	43.0
41–50	15	23.44	20	31.2	35	27.3
51–60	4	6.25	12	18.8	16	12.5
61–70	2	3.1	1	1.6	3	2.35
> 70	1	1.6	–	–	1	0.8
Educational status						
Illiterate	15	23.4	18	28.1	33	25.75
Read and write	12	18.75	17	26.56	29	22.7
Primary	24	37.5	19	29.7	43	33.6
Secondary	13	20.8	10	15.6	23	18.2
Average family size (mean ± SE)	7.89 ± 0.4		7.70 ± 0.38		7.8 ± 0.39	

The educational status of the respondents in the present study was 33.6, 25.75, 2.7, and 18.2% for primary attendants, illiterate, read and write, and secondary attendants, respectively. The result of this study shows that majority of the respondents in the district were literate which has significant importance to adopt new technologies and innovations in to the communities. In contrast to this report, the study Dejen (2010) described that the proportions of illiterate, primary attendants, read and write, and secondary attendants with 33.55, 54.75, 8.35, and 3.35% respectively in southern Ethiopia.

The average family size of the households was 7.8 ± 0.39 (ranging from 2 to 14), which was closer to 8.5 reported for the same district previously (Yoseph et al. 2015). However, the present family size was much higher than the average family sizes (5.9) of the country (CSA 2015) and Benchi Maji and Keffa zone southern Ethiopia (6.7 ± 3) (Dejen 2010). The higher family size in the current study indicates the existence of polygamous marriages and lack of awareness on family planning which requires intervention in this area.

### Landholding and its allocation

The average landholding per household in the study area was 2.13 ha (Table 2). The result was consistent with 2.15 ha reported for the same district previously (Yoseph et al. 2015). The average landholding per household showed a significant difference (*p* < 0.05) between the two agro ecologies of the study area. Accordingly, landholding was higher for highland (2.38 ha ± 0.06) compared to mid-altitude (1.88 ± 0.05) agro ecology. The reason for small land size in mid-altitude agro ecology was mainly due to conduciveness of the area for cash crop production especially for coffee production which attracts more inhabitants in this agro ecology. Landholdings range from 1.01 to 2.00 ha for about 30.8% of farmers in the SNNPR and for 33.3% of farmers at the national level (CACC 2003). The average landholdings per household in Bensa districts was greater than that reported in Gomma district of Jima zone (1.93 ha) (Belete 2009). The size of landholding is an important factor that determines availability of feed for livestock. Thus, feed resources are more available in highland compared to mid-altitude agro ecology in the study area.

**Table 2:** Mean (± SE) landholding per household in Bensa district of Sidama zone

Descriptor	Agro ecology of the study district		
	Highland (*n* = 64)	Mid-altitude (*n* = 64)	Overall mean (*n* = 128)
	Mean ± SE	Mean ± SE	Mean ± SE
Cereal crops	0.5 ± 0.07	0.84 ± 0.07	0.67 ± 0.07
Pulse crop	0.2 ± 0.03	0.06 ± 0.01	0.13 ± 0.02
Sugar cane	0	0.12 ± 0.01	0.06 ± 0.005
Coffee	0	0.3 ± 0.02	0.15 ± 0.023
Enset	0.75 ± 0.07	0.25 ± 0.04	0.5 ± 0.055
Grazing land	0.52. ± 0.05	0.26 ± 0.04	0.39 ± 0.045
Forestland	0.245 ± 0.02	0.05 ± 0.01	0.15 ± 0.015
Vegetables	0.165 ± 0.008	0	0.083 ± 0.004
Average total landholding (ha)	2.38 ± 0.06	1.88 ± 0.05	2.13 ± 0.04

### Farming activities

The major farming activities and their contribution as food and income source to the family in the study area are presented in Table 3. Thus, the major source of food as ranked by the sampled households was cattle production followed by crop and sheep farming, while the major source of cash income for household was both crops and cattle production followed by sheep. Among these crops, enset was used as the main source of food for household consumption, source of income, and for livestock feed in both agro ecology of the study area. On the other hand, maize and haricot bean were the major crops used for income and household consumption in the mid-altitude agro ecology while barley and wheat were the major crops used for income and household consumption in highland agro ecology of the study area. Coffee is an important source of cash particularly in the mid-altitude agro ecology while bean, peas, cabbage and onion were used as additional source of income in highland agro ecology.

**Table 3:** Major farming activities for the supply of food and income in the study area

Importance	Species	Rank			Index
		1st	2nd	3rd	
Food	Cattle	17.2	62.5	7	0.40
	Crop	61.7	21.9	16.4	0.30
	Sheep	13.3	7	63.3	0.20
	Chicken	7.8	8.6	21.9	0.10
Income	Crop	49.2	13.3	29.7	0.35
	Cattle	34.4	49.2	7.8	0.34
	Sheep	16.4	21.1	62.5	0.26
	Chicken	0	7.8	8.6	0.04

Index = sum of (3 × number of household ranked first + 2 × number of household ranked second + 1 × number of household ranked third) given for each variable divided by sum of (3 × number of household ranked first + 2 × number of household ranked second + 1 × number of household ranked third) for all variables

### Livestock species composition

The average livestock holding/household of the study district is presented in Table 4. Respondents in highland had significantly (*p* < 0.05) higher number of cattle, sheep, and horses than respondents in the mid-altitude. However, they had significantly lower (*p* < 0.05) number of chicken, goat, and donkey compared to mid-altitude agro ecology.

**Table 4:** Species composition and livestock holdings in the study area

Descriptor	Highland (*n* = 64)	Mid-altitude (*n* = 64)	Overall mean (*n* = 128)
	Mean ± SE	Mean ± SE	Mean ± SE
Cattle	4.5 ± 0.57^a^	3.0 ± 0.17^b^	3.75 ± 0.37
Sheep	4.6 ± .33^a^	4.3 ± 0.213^a^	4.45 ± 0.27
Goat	0.03 ± 0.2^b^	0.2 ± 0.06^a^	0.1 ± 0.13
Chicken	1.78 ± 0.30^b^	2.9 ± 0.312^a^	2.34 ± .30
Donkey	0.04 ± 0.26^b^	1.8 ± 0.49^a^	0.92 ± 0.37
Horse	0.2 ± 0.55^a^	0.06 ± 0.30^b^	0.13 ± 0.42
Total herd size	11.15 ± 2.2	12.26 ± 1.5	11.7 ± 1.85

^a, b^Superscript letters show significant difference between two agro ecologies

Sheep was the largest livestock species possessed by the two agro ecology of the study area. The possible reasons can be sheep is easy to manage and the area is cereal crop- and vegetable-producing area which is conducive for grazers or sheep production. The average sheep flock size (4.45) per household was by far lower than 31.6 reported for Menz sheep (Getachew et al. 2010, 12.5 reported for North Wollo zone (Tassaw 2012); but higher than 3.6 reported for Goma sheep (Belete 2009). Most of the time cash crop-growing areas have scarcity of land and high population density per square kilometer. These in turn affect the number of livestock per household.

### Sheep flock size and structures

Sheep flock size of the sampled households in the study area is presented in Table 5. The flock owner determines the flock composition on the basis of economic and management considerations. The average sheep flock size in the study area was 4.6 ± 0.33 in highland and 4.3 ± 0.21 in mid-altitude. The higher proportion of females (72.52%) in the flock in the present findings is consistent with sheep flock structure reported for Menz sheep where breeding ewes (49.2%) were dominant (Getachew et al. 2010). Keeping of high proportion of female sheep imply the production of larger number of lambs which has direct impact on selection intensity.

**Table 5:** Average sheep flock structure of the surveyed households in the study area

Age category	Agro ecology of the study district					
	Highland (*n* = 64)		Mid-altitude (*n* = 64)		Overall mean (*n* = 128)	
	Mean ± SE	%	Mean ± SE	%	Mean ± SE	%
Ram lambs < 6 month	0.81 ± 0.12	17.5	0.48 ± 0.07	11	0.64 ± 0.09	14.25
Ram lambs (6–12 month)	0.31 ± 0.07	6.7	0.40 ± 0.06	9.17	0.35 ± 0.07	7.93
Breeding rams > 12 months	0.25 ± 0.06	5.43	0.20 ± 0.06	4.65	0.22 ± 0.06	5.04
Castrates (> 1 year)	0.03 ± 0.02	0.64	0.05 ± 0.03	1.16	0.01 ± 0.03	0.59
Ewe lambs (< 6 months)	0.83 ± 0.11	17.9	1.13 ± 0.08	25.9	0.98 ± 0.1	22
Ewes (6–12 months old)	0.89 ± 0.08	19.2	0.68 ± .08	15.6	0.78 ± 0.08	17.4
Breeding ewes (> 12 months old)	1.56 ± 0.09	33.7	1.42 ± 0.09	32.6	1.49 ± 0.09	33.12
Total flock size	4.6 ± 0.08				4.3 ± 0.07	

### Feeds and feeding systems

The quality and quantity of feed resources available for animals primarily depend upon the climatic and seasonal factors (Zewdu 2008). Feed resources commonly used by farmers in the study area across the different seasons are presented in Table 6. The major feed resources for sheep during the wet season were natural pasture followed by crop residues across in the two agro ecologies. The findings of the current study (wet season feed availability) in Bensa district of Sidama zone was similar with Tesfaye (2009), Grum (2010), and Amelmal (2011) who reported for Metema district of Amhara region, around Dire Dawa, Dawuro Zone, and Konta Special Woreda of SNNPR, respectively. However, the major feed resources during the dry season across the two agro ecologies were crop restudies followed by natural pasture. Enset leaf and stem, and bamboo leaf are also important feed resources used to complement feed supply particularly during the dry season when the availability of forage is low.

**Table 6:** Major feed resources of sheep during the dry and wet seasons in the study area

Feed Resources	Wet season								Dry season							
	Highland					Mid-altitude			Highland					Mid-altitude		
	1st	2nd	3rd	Index	1st	2nd	3rd	Index	1st	2nd	3rd	Index	1st	2nd	3rd	Index
Natural pasture	100	–	–	0.54	100	–	–	0.62	15.2	50	23.4	0.28	37.5	43.8	10.9	0.36
Hay	–	–	–	0	–	–	–	0	–	6.2	14.1	0.04	–	–	–	0
Crop residues	–	95.3	–	0.34	–	92.2	–	0.37	45.42	27.8	6.5	0.33	50	40.6	–	0.40
Fallow land	–	–	42.2	0.08	–	1.6		0.01	1.6	–	25	0.05	3.1	14.1	1.6	0.07
Concentrates	–	–	3.1	0.01	–	–	–	0	–	–	–	0	–	–	21.9	0.04
Enset leaf and stem	–	–	18.8	0.03	–	–	–	0	23.4	6.2	25	0.18	–	1.6	26.9	0.05
Bamboo leaf	–	–	–	–	–	–	–	–	14.5	9.7	6	0.12	–	–	–	0
Sugar cane tops	–	–	–	–	–	–	–	–	–	–	–	–	9.4	–	20	0.08

Index = sum of (3 × number of household ranked first + 2 × number of household ranked second +1 × number of household ranked third) given for each variable divided by sum of (3 × number of household ranked first +2 × number of household ranked second +1 × number of household ranked third) for all variables

The major crop residue in highland agro ecology includes barely, wheat, bean, and peas straws while in mid-altitude, it includes maize stover, wheat, haricot bean, and teff straws.

The difference in type of crop residues availability between the two agro-climates is due to difference in agro-climatic requirements of the different crops.

### Watering resource and utilization

Table 7 showed that river water was the major water source of sheep in wet and dry seasons in both agro ecologies. The proportion of sheep watered by river water were 78.12 and 75% during the dry and wet seasons, respectively, in highland agro-climate while it was 65.62 and 60.9% during the wet and dry seasons, respectively, in mid-altitude agro-climate. The distances to watering points varied during the dry and wet seasons.

**Table 7:** Water sources and utilization during dry and wet seasons

Descriptors	Dry season				Wet season			
	Highland		Mid-altitude		Highland		Mid-altitude	
	*N*	%	*N*	%	*N*	%	*N*	%
Source of water								
River	50	78.12	42	65.62	48	75.0	39	60.9
Spring	14	21.88	22	34.38	15	23.4	25	39.1
River and spring	–	–			1	1.6	–	–
Distance of water								
Water at home	5	7.8	7	10.9	8	12.5	14	21.9
Less than 1 km	49	76.56	40	62.5	51	79.68	38	59.4
1–5 km	10	15.6	14	21.87	5	7.8	11	17.2
6–10 km			3	4.68			1	1.6
Greater than 10 km								
Frequency of water								
Free available	8	12.5	9	14.1	4	6.25	10	15.6
Once a day	52	81.25	55	85.9	54	84.37	53	82.8
Once every 2 days	4	6.25	–	–	6	9.37	1	1.6

The majority (76.5% for dry and 79.6% for wet seasons) of the respondents water their animals within less than 1-km distance in highland agro-climate. Similarly, majority of the households (62.5% for dry and 59.4% for wet seasons) water their animals at less than 1 km in mid-altitude agro-climate. Similar to this study, Workneh and Rowlands (2004) reported that the majority of households (three-fourth) water their animals with less than 1 km in wet season Oromia region. In the contrary study, Hulunim (2014) described that 45.45 and 23.48% of households in Borena and Siti (around Dire Dawa) areas traveled more than 6 km to find water. The result of this study shows that a few respondents indicate more than 5-km travel in search of water for their livestock. Hence, in the study area, there was no scarcity of water or it was not a limiting factor for sheep production.

### Housing of sheep

In the study area, different types of houses, housing materials, and the common housing systems were identified (Table 8). The majority of the respondent in both agro ecologies house their sheep in the main house together with the family. Separate sheep house with roof was also reported by some farmers across two agro ecologies. The majority of the farmers in the study district house their sheep during the night. The majority of respondents house their sheep together with cattle while 3.1% of house separately.

**Table 8:** Housing of sheep in the study area

Type of housing	Agro ecology					
	Highland		Mid-altitude		Overall total	
	*N*	%	*N*	%	*N*	%
Family house with roof	47	73.4	50	78.12	97	75.76
Separate house with roof	17	26.6	14	21.88	31	24.24
Type of housing material						
Grasses or bushes	55	85.9	58	90.6	113	88.25
Grass/bushes	9	14.1	6	9.4	15	11.75
Sheep is housed						
Separately	2	3.1	–	–	2	1.55
Together with cattle	62	96.9	64	100		98.45

### Major diseases and parasites of sheep

Diseases have numerous negative impacts on livestock herds and flocks; it causes death of animals, loss of weight, slow down growth, poor fertility performance, and decrease in physical power (CSA 2012). Healthy sheep with normal physiological function and structure that enable the sheep to attain highest production is vital. Farmers in the study area do not exactly know the type of disease which causes mortality, but they were able to describe the symptoms. According to the livestock and fishery office of Bensa district, the major types of diseases and parasites of sheep which were frequently occurred in the study area are presented in Table 9. Accordingly, *Ovine pasteurellosis* (Sonbe), contagious caprine pleuropneumonia (Shonbe), and sheep pox were the major diseases, while liver fluke and external parasites were the major parasites prevalent in the area. The cold environment prevailing in the survey district might have predisposed the animals to respiratory diseases such as pasteurollosis. Similar diseases were reported across different part of the country by different authors (Zewdu 2008) in Keffa Zone of southern Ethiopia; Tesfaye 2008 in Menz of Ethiopia). According to focus group discussants, most of the respondents in the study area use modern veterinary drugs to treat sick animals and it were entirely dependent on government service since there was no private veterinary service provider. In general, for effective breeding strategy, sheep producers should be encouraged to adopt proper and cost effective disease control measures, and the limited animal health services need to be strengthened.

**Table 9:** Major sheep diseases and parasites in the study area

Common name	Local name (Sidamgna)	Season of occurrence	Susceptible age group	Rank
Diseases and parasites				
Ovine pasturiolosis	Shonbe	Any time of the year	All age group	1
CCPP	Sonbe	Any time of the year	All age group	2
Sheep pox	Fexelle	Any time of the year	All age group	5
Liver fluke	Gognogne	Any time of the year	All age group	3
External parasite	Mejabinno	During dry season	All age group	4

*CCPP* contagious caprine pleuropneumonia

### Reproductive performance of indigenous sheep

Good reproductive performance is a prerequisite for any successful sheep production program. Reproductive performances of sheep in the study area are presented in Table 10. There was significant (*p* < 0.05) difference between the agro ecologies with respect to the reproductive performance of indigenous sheep because of the presence of better sheep management practices in the highland than the mid-altitude which is related with large size of grazing land in the highland. The average age at sexual maturity of male sheep was 7.07 months. Similarly, an average age of 7.1 months was reported for Afar sheep earlier (Tesfaye 2008). Average age at sexual maturity of male sheep was earlier than that of female counterpart. The average age at first lambing observed in both agro ecologies was smaller than 14.77 ± 1.8 months reported for Dawuro and Konta special woreda sheep (Amelmal 2011). Better reproductive performance observed in this study may indicate the sheep management, good husbandry practices of the community, and the suitability of the study area for sheep production.

**Table 10:** Reproductive performances of indigenous sheep population in the study area

Reproductive parameters	Agro ecology of the study district					
	Highland		Mid-altitude		Over all mean	
	*N*	Mean ± SE	*N*	Mean ± SE	*N*	Mean ± SE
Average age at sexual maturity of (male; month)	64	7 ± 0.12^b^	64	7.15 ± 0.2^a^	128	7.07 ± 0.16
Average age at sexual maturity (female; month)	64	7.68 ± 0.23^b^	64	7.8 ± 0.12^a^	128	7.74 ± 0.175
Age at first lambing	64	12.16 ± 0.27^b^	64	13.5 ± 0.21^a^	128	12.84 ± 0.24
Lambing interval	64	9.38 ± 0.07^b^	64	9.8 ± 0.14^a^	128	9.59 ± 0.1
Reproductive life time of ewes (years)	64		64	7.86 ± 0.06^b^	128	8.1 ± 0.11
Average number of lambs per ewes life time	64	9.1 ± 0.23^a^	64	8.28 ± 0.14^b^	128	8.69 ± 0.185
Average number of lambs per lambing	64	1.3 ± 0.34^a^	64	1.2 ± 0.15^b^	128	1.25 ± 0.24

Different superscripts denote significant differences at *p* < 0.05 between means of the agro ecologies

*n* number HH, *SE* standard error

The result of the present study was in agreement with the studies Amelmal (2011) and Zewdu (2008) that indicated 10.33 for Marka and 8.9 lambing interval for Bonga sheep, respectively. The finding of the present study on life time lamb crop is slightly lower than 9.42 reported by Amelmal (2011) for Dowuro zone and Konta special woreda sheep. Productions of large number of progeny in ewe’s life span provide ample scope for selection and genetic improvement other than large numbers of animals for sale. The observed value was higher than the litter size of East African sheep under pastoral management systems were reported in the range of 1.03, 1.05, and 1.14 in Ethiopia, Kenya, and Sudan, respectively (Wilson 1982) but lower than sheep breed (50.1%) in Bench-Maji Zone (Dejen 2010).

### Flock dynamics

Knowledge about ways of acquisition of breeding stock and mode of disposal is important in assessing the breeding practices of sheep owners (Helen et al. 2015). Major modes of flock entry and exit are summarized in Table 11. In the study area, sheep were added into the farm through birth, purchase, and exchange, of which the contribution of the former (87.5%) was the highest followed by purchase (8.55%). The contribution of exchange as source of animals was very minimal (3.9%). Similarly, birth was reported as the main mode of indigenous sheep flock entry in Horro, Adiya Kaka, and Alaba districts (Tsedeke 2007; Zewdu 2008). The highest share of the total exit (70.85%) was accounted for sale, followed by mortality (11.9%) and exchange (10%) while only 7.25% was reportedly slaughtered.

**Table 11:** Mode of sheep flock entry and exit (in percent) during the last 12 months

Means	Agro ecology		
	Highland	Mid-altitude	Overall total
Entry			
Birth	89.1	86	87.55
Purchase	6.2	10.9	8.55
Exchange	4.7	3.1	3.9
Exit			
Sale (off take)	70.2	71.5	70.85
Death	11.4	12.4	11.9
Exchange	10.4	9.6	10
Slaughter	8	6.5	7.25

### Constraints for sheep production

Participatory identification and prioritization of the major constraints of livestock production is the first step to design and implement need-based interventions development options. Constraints impending sheep productivity in the study area are presented in Table 12. Although the major constraints limiting sheep breeding were mostly similar, their importance, however, varied across the study areas. This study observed that feed shortage, disease, genotype, and market were the major constraints challenging sheep production across both agro ecologies. Feed shortage have been reported by the majority of respondents as common constraint and ranked first. Similar results were reported for Menz and Afar areas (Tesfaye 2008). The major causes of feed scarcity were shortage of grazing land and expansion of arable farming at the expense of grazing land.

**Table 12:** Ranking of sheep production constraints by smallholder farmers (%)

Constraints	Agro ecology							
	Highland				Mid-altitude			
	1st rank	2nd rank	3rd rank	Index	1st rank	2nd rank	3rd rank	Index
Genotype	7.8	18.8	35.9	0.16	26.6	14.1	26.6	0.23
Feed shortage	45.3	10.9	6.2	0.28	53.1	18.8	10.9	0.35
Disease	9.4	42.2	18.8	0.22	6.2	43.8	25	0.21
Market	17.2	12.5	21.9	0.17	14.1	14.1	29.7	0.165
Predator	4.7	1.6	–	0.03	–	1.6	–	0.005
Labor	15.6	14.1	3.1	0.13	1.6	4.7	3.1	0.03

Predator and labor problem were among the minor reported problems limiting sheep farming across the study areas. However, labor shortage received a little bit higher proportion around highland area as compared with mid-altitude. Therefore, since producers across all the study areas practice traditional sheep farming, awareness should be created on the merits of sheep improvement before implementations of breed improvement programs. In the present study, ranking of sheep rearing constraints (indices) by the producer’s reflect their priority needs for intervention. Hence, governmental and nongovernmental organizations should give diligent attention to address the problems according to its importance.

## Conclusion and recommendations

It was observed from the above study that the sheep production system in the study area was more of extensive production system. Hence, for enhancing future production from sheep in the area, emphasis is to be given on feed availability, disease management, breeding policy, and marketing strategies.

Based on the current study, the following recommendations have been made:

- Since feed shortage in terms of quantity and quality is among the leading constraints limiting sheep value chain development in the study area, efforts should be made to improve grazing land through top dressing with urea and controlled grazing, introduction of improved fodder grasses and legumes consistent with the respective farming system, and enhancement of the nutritive value of crop residues through urea treatment.
- Prevalence of disease and parasites and poor health management negatively influenced productivity of sheep flock in the study area. Hence, the type, seasonal occurrence, and economic loose due to the diseases and parasites should be documented and pertinent control measure should be introduced.
